# Synthesis and Biological Evaluation of New 1,3-Thiazolidine-4-one Derivatives of 2-(4-Isobutylphenyl)propionic Acid

**DOI:** 10.3390/molecules190915005

**Published:** 2014-09-18

**Authors:** Ioana Mirela Vasincu, Maria Apotrosoaei, Andreea-Teodora Panzariu, Frédéric Buron, Sylvain Routier, Lenuta Profire

**Affiliations:** 1 Department of Pharmaceutical Chemistry, Faculty of Pharmacy, University of Medicine and Pharmacy “Grigore T. Popa”, 16 University Street, Iasi 700115, Romania; E-Mails: ioanageangalau@yahoo.com (I.M.V.); mariasutu@yahoo.com (M.A.); teutz_panzariu@yahoo.com (A.-T.P.); 2 Institute of Organic and Analytical Chemistry, University of Orléans, Orléans 45076, Cedex 2, France; E-Mail: frederic.buron@univ-orleans.fr

**Keywords:** ibuprofen, thiazolidine-4-one, synthesis, spectral methods, antioxidant effect

## Abstract

New thiazolidine-4-one derivatives of 2-(4-isobutylphenyl)propionic acid (ibuprofen) have been synthesized as potential anti-inflammatory drugs. The structure of the new compounds was proved using spectral methods (FR-IR, ^1^H-NMR, ^13^C-NMR, MS). The *in vitro* antioxidant potential of the synthesized compounds was evaluated according to the total antioxidant activity, the DPPH and ABTS radical scavenging assays. Reactive oxygen species (ROS) and free radicals are considered to be involved in many pathological events like diabetes mellitus, neurodegenerative diseases, cancer, infections and more recently, in inflammation. It is known that overproduction of free radicals may initiate and amplify the inflammatory process via upregulation of genes involved in the production of proinflammatory cytokines and adhesion molecules. The chemical modulation of acyl hydrazones of ibuprofen **3a**–**l** through cyclization to the corresponding thiazolidine-4-ones **4a**–**n** led to increased antioxidant potential, as all thiazolidine-4-ones were more active than their parent acyl hydrazones and also ibuprofen. The most active compounds are the thiazolidine-4-ones **4e**, **m**, which showed the highest DPPH radical scavenging ability, their activity being comparable with vitamin E.

## 1. Introduction

Ibuprofen, a nonsteroidal anti-inflammatory drug (NSAID) with an arylpropionic acid structure is very useful in therapy for its analgesic, antipyretic and anti-inflammatory effects [1,2]. Long-term use of this drug, like most NSAIDs, has however been associated with gastro-intestinal (GI) ulceration, bleeding and nephrotoxicity [3,4]. The anti-inflammatory effects of NSAIDs are related to the inhibition of prostaglandin synthesis by blocking the activity of cyclooxygenase enzyme (COX) [5]. It is known that COX exists in two isoforms, constitutive COX-1 that provides cytoprotection in the gastrointestinal tract and inducible COX-2, which mediates inflammation [5,6]. The GI damage induced by NSAIDs is generally attributed to two factors, *i.e.*, local irritation caused by the carboxylic acid moiety common to most NSAIDs (topical effect) and decreased production of tissue-cytoprotective prostaglandins [7]. Although selective COX-2 inhibitors are very efficient as anti-inflammatory agents and safer to gastrointestinal tract, recent data showed that therapy with COX-2 selective inhibitors could be associated with increased cardiovascular risks [8]. In order to improve the anti-inflammatory effect and safety profile of representative NSAIDs, one research strategy is derivatization of the carboxylic acid group with various heterocyclic systems (oxazole, izoxazole, pyrazole, oxadiazole, thiazole, thiadiazole, triazole, *etc.*) [9,10]. In the past two decades there has been considerable interest in the role of reactive oxygen species (ROS) in inflammation [11]. ROS mediate the oxidative degradation of cellular components and alteration of protease/antiprotease balance with damage to the corresponding tissue. In the early stages of the inflammatory process, ROS exert their actions through activation of nuclear factors, such as NFkB or AP-1, that induce the synthesis of cytokines. In later stages, endothelial cells are activated due to the synergy between free radicals and cytokines, promoting the synthesis of inflammatory mediators and adhesion of molecules. In the last step free radicals react with different cellular components (trypsin, collagen, LDL, DNA, lipids) inducing the death of cells [12,13].

The thiazolidine-4-one moiety is a heterocycle that has received more attention in the last years due its important biological properties [14]. Many effects have been found, including anti-inflammatory and analgesic [15], antitubercular [16], antimicrobial and antifungal [17], antiviral, especially as anti-HIV agents [18], anticancer, antioxidants [19], anticonvulsants [20] and antidiabetic activity [21]. In the present study, some new derivatives of ibuprofen that contain thiazolidine-4-one scaffolds were synthesized in order to obtain compounds with double effect—antioxidant and anti-inflammatory properties. The structures of the compounds were assigned based on their spectral data (FT-IR, ^1^H-NMR, ^13^C-NMR, MS) and the compounds were screened for their *in vitro* antioxidant potential.

## 2. Results and Discussion

### 2.1. Chemistry

**Scheme 1:**
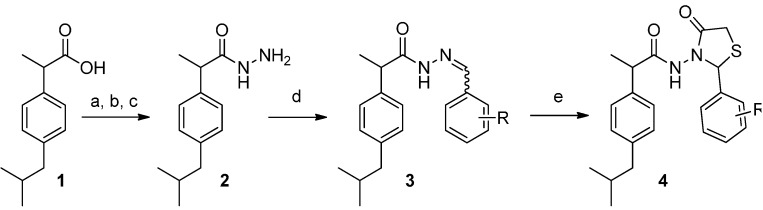
Synthetic procedure of compounds **4a**–**n**.

The 1,3-thiazolidine-4-one derivatives **4a**–**m** were synthesized in several steps using the method summarized in Scheme 1 and Table 1. First 2-(4-isobutylphenyl)propionic acid (ibuprofen, **1**) was reacted with thionyl chloride, followed by treatment with dry ethanol to get 2-(4-isobutylphenyl)propionic acid ethyl ester, which was turned in 2-(4-isobutylphenyl)propionic acid hydrazide (**2**) by reaction with 66% hydrazine hydrate [22]. The condensation of compound **2** with various aromatic aldehydes allowed the preparation of the corresponding hydrazone derivatives **3a**–**l** in satisfactory yields. Finally, the hydrazone derivatives of ibuprofen upon reaction with mercaptoacetic acid led to the thiazolidine-4-one derivatives **4a**–**l** in moderate to good yields. By reduction of compound **4g** in presence of tin chloride and few drops of acetic acid in ethanol_,_ the thiazolidine-4-one **4m** was obtained in 90% yield. Acetylation of **4m** with acetyl chloride gave thiazolidine-4-one **4n **in moderate yield.

*Reagents and conditions*: (**a**) thionyl chloride, reflux, 4 h; (**b**) dry ethanol, reflux, 4 h; (**c**) hydrazine hydrate, dry ethanol, reflux, 12 h; (**d**) aromatic aldehyde, dry ethanol, reflux, 4 h; (**e**) mercaptoacetic acid, heating 2–8 h.

**Table 1:** Synthesized derivatives **3** and **4**.

Entry	Product 3	N°, Yield	Product 4	N°, Yield
1	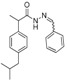	**3a**, 76%	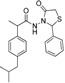	**4a**, 57%
2	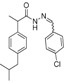	**3b**, 91%	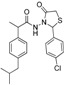	**4b**, 76%
3	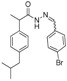	**3c**, 93%	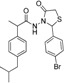	**4c**, 77%
4	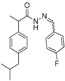	**3d**, 84%	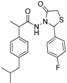	**4d**, 66%
5	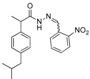	**3e**, 91%	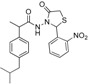	**4e**, 73%
6	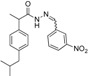	**3f**, 85%	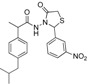	**4f**, 62%
7	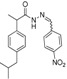	**3g**, 69%	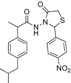	**4g**, 72%
8	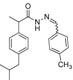	**3h**, 86%	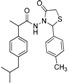	**4h**, 41%
9	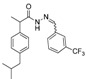	**3i**, 69%	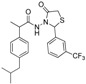	**4i**, 53%
10	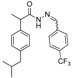	**3j**, 69%	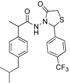	**4j**, 38%
11	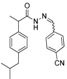	**3k**, 52%	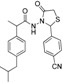	**4k**, 87%
12	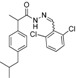	**3l**, 60%	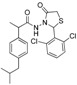	**4l**, 38%
13	-	-	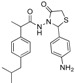	**4m**, 90%^a^
14	-	-	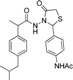	**4n**, 47%^b^

**4g**, SnCl_2_, acetic acid, ethyl alcohol, room temperature 14 h; **4m**, NaH, acetyl chloride, DMF, reflux, 14 h.

The structure of the compounds was assigned on the basis of their spectral data (IR, ^1^H-NMR, ^13^C-NMR, MS) which are provided in the Experimental Section. The spectral data of compound **2** was in agreement with the literature [23]. The IR spectra of the acyl hydrazone derivatives of ibuprofen **3a**–**l** showed characteristic absorption bands corresponding to their NH, C=O and C=N groups at 3185–3180 cm^−1^, 1669–1662 cm^−1^ and 1608–1596 cm^−1^, respectively. The appearance of the stretching band of the thiazolidine-4-one C=O at 1710–1721 cm^−1^, together with the characteristic C-S absorption band at 656–694 cm^−1^ confirmed the formation of compounds **4a**–**n**.

In the ^1^H-NMR spectra of compounds **3a**–**l** all groups exhibited two sets of signals. It is known that N-acylhydrazones can exist in four possible forms, as geometrical isomers (*E*/*Z*) with respect to the C=N double bond and as rotamers (*cis/trans*) due to the amide N-C(O) bond [24]. Based on literature data the N-acylhydrazones derived from aromatic aldehydes are in the *E* form because the Z*_N-N_* conformer is not formed due to the steric hindrance on the imine bond [25]. All compounds were found to exist as racemic mixtures of two isomers, as indicated by their ^1^H-NMR spectra. The two sets of signals indicate the possibility of equilibrium and interconversion between rotamers (and/or configurational isomers) in solution. The signals of the azomethine group (N=CH) proton of one form appeared at δ 8.17–8.61 ppm, while the proton of the other form appeared at δ 7.89–8.30 ppm as a singlet. The NH proton (CO-NH-N=) of one form also appeared at δ 11.44–11.79 ppm, whereas the proton of the other form appeared at δ 11.18–11.55 ppm as a singlet. In the ^13^C-NMR spectra of the compounds **3a**–**l** the azomethine carbon (N=CH) of one form resonated at δ 141.76–146.94 ppm whereas the carbon of the other form appeared at δ 137.97–142.92 ppm. The formation of the thiazolidine-4-one heterocyclic system has been proved by the characteristic NMR data. In the ^1^H-NMR spectra of the compounds **4a**–**n** the CH(SCHN) protons resonate as a singlet between 5.66–6.71 ppm and 5.46–6.57 ppm corresponding to the isomer forms. The protons of the methylene group (-CH_2_-S) appears as multiplets or doublets of doublets between 3.61–3.97 ppm and 3.70–3.77 ppm. The carbons of the thiazolidine-4-one system appear in the ^13^C-NMR spectra between 55.72–61.80 ppm and 28.45–29.64 ppm. The proton and carbon signals for other characteristic groups were all observed according to the expected chemical shift and integral values. The NMR spectral data, coupled with the mass spectra, strongly support the proposed structures of the all synthesized compounds.

### 2.2. Biological Evaluation

#### 2.2.1. Total Antioxidant Activity

The total antioxidant activity was determined measuring the phophomolybdenum blue complex with a maximum absorption at 695 nm [26]. The data, which are presented in Table 2 and Table 3, showed that all the tested compounds are more active than ibuprofen. It was also observed that the total antioxidant activity of acyl hydrazone derivatives **3a**–**l** (Table 2) was improved by cyclization to the corresponding thiazolidine-4-ones **4a**–**l** (Table 3) which supports the favorable influence of thiazolidin-4-one scaffold for antioxidant potential.

**Table 2:** Total antioxidant activity (EC_50_ µg/mL) of the acylhydrazone derivatives **3a**–**l**.

Sample	EC_50_ µg/mL	Sample	EC_50_ µg/mL
**3a**	79.70 ± 0.70	**3g**	78.42 ± 1.34
**3b**	71.96 ± 1.12	**3h**	70.10 ± 1.14
**3c**	96.78 ± 1.22	**3i**	84.57 ± 1.28
**3d**	77.07 ± 0.51	**3j**	106.19 ± 1.64
**3e**	82.39 ± 0.83	**3k**	68.01 ± 1.72
**3f**	73.32 ± 0.32	**3l**	91.64 ± 1.68
Ibuprofen	773.67 ± 3.41	Vitamin E	26.18 ± 0.51

Data are mean ± SD (n = 3, *p* < 0.05).

**Table 3:** Total antioxidant activity (EC_50_ µg/mL) of the thiazolidine-4-one derivatives **4a**–**n**.

Sample	EC_50_ µg/mL	Sample	EC_50_ µg/mL
**4a**	73.58 ± 0.96	**4h**	74.02 ± 0.97
**4b**	53.98 ± 1.26	**4i**	96.17 ± 1.48
**4c**	82.26 ± 0.41	**4j**	58.84 ± 1.47
**4d**	74.14 ± 1.39	**4k**	74.20 ± 0.50
**4e**	76.16 ± 1.03	**4l**	60.83 ± 0.86
**4f**	70.04 ± 1.29	**4m**	85.10 ± 0.21
**4g**	53.46 ± 0.90	**4n**	109.85 ± 2.56
Ibuprofen	773.67 ± 3.41	Vitamin E	26.18 ± 0.51

Data are mean ± SD (n = 3, *p* < 0.05).

The most active compounds were **4b** (EC_50_ = 53.98 ± 1.26), **4g** (EC_50_ = 53.46 ± 0.90), **4j** (EC_50_ = 58.84 ± 1.47) and **4l** (EC_50_ = 60.83 ± 0.86) which contain Cl(4), NO_2_(4), CF_3_(3) and 2,6-diCl as substituents on the thiazolidine-4-one aromatic ring. These compounds were about 14 times (**4b**, **4g**) and 13 times (**4j**, **4l**) more active than ibuprofen (EC_50_ = 773.67 ± 3.41), respectively. Important antioxidant activity has also been shown by the compounds **4d **(EC_50_ = 70.04 ± 1.29), **4a** (EC_50_ = 73.58 ± 0.96), **4h **(EC_50_ = 74.02 ± 0.97), **4d **(EC_50_ = 74.14 ± 1.39), **4k** (EC_50_ = 74.20 ± 0.50) and **4e** (EC_50_ =76.16 ± 1.03) that are 10–11 times more active than parent compound ibuprofen. The tested compounds were however less active than vitamin E used as positive control.

#### 2.2.2. DPPH Radical Scavenging Assay

DPPH is a well-known radical used as indicator to evaluate the radical scavenging ability of antioxidants. In methanol solution DPPH has an intense violet color with a UV-Vis absorption band at 517 nm, and it becomes pale yellow or colorless when neutralized in the presence of proton donating agents [27]. The DPPH radical scavenging ability (%) of samples at concentrations of 487.8 µg/mL is presented in Table 4 and Table 5. Higher values of scavenging ability indicate a higher radical scavenging effectiveness potential.

**Table 4:** The DPPH radical scavenging ability of the acyl hydrazone derivatives **3a**–**l**.

Sample	Scavenging Ability (%)	Sample	Scavenging Ability (%)
**3a**	4.19 ± 0.22	**3g**	1.25 ± 0.13
**3b**	5.13 ± 0.61	**3h**	6.80 ± 0.55
**3c**	5.14 ± 0.47	**3i**	4.24 ± 0.55
**3d**	6.27 ± 0.45	**3j**	5.57 ± 0.62
**3e**	17.57 ± 0.21	**3k**	2.16 ± 0.15
**3f**	13.52 ± 0.20	**3l**	5.82 ± 0.55
Ibuprofen	4.15 ± 0.22	Vitamin E *	96.48 ± 0.94

* 0.25 mg/mL stock solution; Data are mean ± SD (n = 3, *p* < 0.05).

**Table 5:** The DPPH radical scavenging ability of the thiazolidine-4-one derivatives **4a**–**n**.

Sample	Scavenging Ability (%)	Sample	Scavenging Ability (%)
**4a**	59.64 ± 0.28	**4h**	9.89 ± 0.29
**4b**	81.31 ± 0.55	**4i**	17.85 ± 0.17
**4c**	7.98 ± 0.32	**4j**	3.02 ± 0.09
**4d**	11.53 ± 0.18	**4k**	53.29 ± 0.26
**4e**	94.42 ± 0.43	**4l**	10.62 ± 0.17
**4f**	43.33 ± 0.65	**4m**	94.88 ± 0.57
**4g**	67.99 ± 0.60	**4n**	11.66 ± 0.17
Ibuprofen	4.15 ± 0.22	Vitamin E *	96.48 ± 0.94

* 0.25 mg/mL stock solution; Data are mean ± SD (n = 3, *p* < 0.05).

In the acyl hydrazone series most of the the tested compounds showed a radical scavenging ability comparable with ibuprofen (Table 4). The most active compounds were **3e** and **3f** which are about three times and two times more active than their parent compound, respectively. The scavenging ability of the acyl hydrazones was improved by cyclization to the corresponding thiazolidine-4-one derivatives, these compounds all being more active than ibuprofen, except for compound **4j** which contains a CF_3_ group in the *meta* position of phenyl ring (Table 5). The most active compounds were **4e** and **4m** which contain NO_2_ and NH_2_ groups in *ortho* and *para* position of the phenyl ring, respectively. For these compounds the radical scavenging ability (%) was 94.42 ± 0.43 and 94.88 ± 0.57, which means that the compounds are about 23 times more active than ibuprofen (4.15 ± 0.22). The activity of these compounds is comparable with that of vitamin E used as positive control. Important radical scavenging ability was also shown by compound **4b** (81.31 ± 0.55), which contains a Cl group in the *para* position of the phenyl ring, the compound being 20 times more active than ibuprofen.

#### 2.2.3. ABTS Radical Scavenging Assay

The ABTS (2,2'-azino-bis(3-ethylbenzothiazoline-6-sulfonic acid) radical cation decolorization assay is based on the ability of hydrogen donating antioxidants to scavenge the long-life radical cation ABTS^+^. ABTS^+^, which is a blue chromophore, is generated by the reaction between 2,2'-azino-bis(3-ethylbenzothiazoline-6-sulfonic acid) and ammonium persulfate. The antioxidant compound produces a discoloration of the solution with a decrease in the absorbance measured at 734 nm [28]. The ABTS radical scavenging ability (%) of samples at concentrations of 200 µg/mL is presented in Table 6 and Table 7. Higher values of scavenging ability indicate a higher radical scavenging effectiveness potential.

**Table 6:** The ABTS radical scavenging ability of the acyl hydrazone derivatives **3a**–**l**.

Sample	Scavenging Ability (%)	Sample	Scavenging Ability (%)
**3a**	7.03 ± 0.35	**3g**	1.67 ± 0.35
**3b**	5.44 ± 0.65	**3h**	13.31 ± 0.81
**3c**	3.21 ± 0.18	**3i**	5.79 ± 0.48
**3d**	4.59 ± 0.37	**3j**	6.07 ± 0.23
**3e**	8.19 ± 0.17	**3k**	3.79 ± 0.35
**3f**	3.69 ± 0.07	**3l**	3.01 ± 0.27
Ibuprofen	4.42 ± 0.18	Vitamin E *	96.75 ± 0.40

* 1 mg/mL stock solution; Data are mean ± SD (n = 3, *p* < 0.05).

**Table 7:** The ABTS radical scavenging ability of the thiazolidine-4-one derivatives **4a**–**n**.

Sample	Scavenging Ability (%)	Sample	Scavenging Ability (%)
**4a**	7.24 ± 0.30	**4h**	7.70 ± 0.57
**4b**	52.17 ± 1.20	**4i**	4.84 ± 0.43
**4c**	4.29 ± 0.21	**4j**	3.44 ± 0.16
**4d**	22.88 ± 0.43	**4k**	51.53 ± 1.29
**4e**	52.79 ± 1.28	**4l**	2.41 ± 0.30
**4f**	10.94 ± 0.51	**4m**	25.48 ± 0.49
**4g**	37.14 ± 1.10	**4n**	10.08 ± 0.39
Ibuprofen	4.42 ± 0.18	Vitamin E *	96.75 ± 0.40

* 1 mg/mL stock solution; Data are mean ± SD (n = 3, *p* < 0.05).

The acyl hydrazone derivatives showed an antioxidant activity comparable with ibuprofen. The most active compound in this series was **3h**, with radical scavenging activity of 13.31 ± 0.81, which means that this compound is three times more active than ibuprofen (4.42 ± 0.18). In the thiazolidine-4-one series the most active compounds were **4b**, **4e** and **4k**, which contain Cl(4), NO_2_(2) and CN(4), respectively, as substituents on the phenyl ring. These compounds, which showed a scavenging ability of around 50%, are 12 times more active than ibuprofen. In comparison with the corresponding acyl hydrazones **3b**, **3e** and **3k** the thiazolidine-4-ones were 10 times (**4b**), seven times (**4e**) and 13 times (**3k**) more active. The improved antiradical activity of acyl hydrazones by cyclization to form thiazolidine-4-ones was also observed for compounds **3d**, **3f** and **3g**. The most favorable influence was observed for acyl hydrazone **4g**, which contains a NO_2_ in the *para* position of the phenyl ring. The corresponding thiazolidine-4-one (**4g**, 37.14 ± 1.10) is 22 times more active than **3g** (1.67 ± 0.35). These data strongly support the favorable influence of the thiazolidine-4-one ring on the antioxidant potential of these compounds. The tested compounds were less active than vitamin E.

## 3. Experimental Section

### 3.1. General Procedures

The melting points were measured using a Buchi Melting Point B-540 apparatus and they are uncorrected. The FT-IR spectra were recorded on Horizon MB^TM^ FT-IR, over a 500–4000 cm^−1^ range, after 32 scans at a resolution of 4 cm^−1^. The spectra processing was carried out with the Horizon MB^TM^ FTIR Software. The ^1^H-NMR (400 MHz) and ^13^C-NMR (101 MHz) spectra were obtained on a Bruker Avance 400 MHz spectrometer using tetramethylsilane as internal standard and DMSO-*d*_6_ as solvent. The chemical shifts were shown in δ values (ppm). The mass spectra were registered using a Bruker MaXis Ultra-High Resolution Quadrupole Time-of-Flight Mass Spectrometer. The progress of the reaction was monitored on TLC, using pre-coated Kieselgel 60 F254 plates (Merck, Whitehouse Station, NJ, USA) and the compounds were visualized using UV light. E-factor and material efficiency (ME) have been selected to evaluate the greenness of the synthetic procedures. E-factor is a very useful metric tool that is defined as E-Factor = mass of wastes/mass of product [29,30]. The E-factor can be used to calculate the material efficiency of the process according to the equation: ME = 1/E-factor +1 [30].

### 3.2. Synthetic Procedures

#### 3.2.1. Preparation of the Acyl Hydrazones **3a**–**l**

Preparation of the acyl hydrazones **3a**–**l** was realized according to a conventional method from the literature [31]. To a solution of 2-(4-isobutylphenyl)propionic acid hydrazide **5** (13.6 mmol) in dry ethanol (50 mL) aromatic aldehydes (13.6 mmol) were added. The reaction mixtures were heated at 90–95 °C until completion of the reaction (TLC monitoring using dichloromethane-methanol, 9.8:0.2, v/v, UV light at 254 nm). The mixture was cooled to room temperature and the solvent was removed by rotary evaporator. The residue was purified on a silica gel column using dichloromethane-methanol (9.8:0.2, v/v) as eluent system. FT-IR and physical data for **3a**–**g** were reported in our previous paper [22].

*2-(4-Isobutylphenyl)propionic acid (benzylidene)hydrazide* (**3a**). 50/50 racemic mixture. ^1^H-NMR (δ ppm): 11.50/11.25 (s, 1H, -NH-N=), 8.21/7.92 (s, 1H, -N=CH-), 7.76–7.56 (m, 2H, Ar-H ), 7.41 (d, *J* = 6.9 Hz, 3H, Ar-H), 7.27 (dd, *J* = 8.0, 4.8 Hz, 2H, Ar-H), 7.08 (dd, *J* = 14.6, 8.0 Hz, 2H, Ar-H), 4.65/3.67 (q, *J* = 6.9 Hz, 1H, -CH-CH_3_), 2.38 (dd, *J* = 13.8, 6.6 Hz, 2H, -CH_2_-CH(CH_3_)_2_), 1.85–1.69 (m, -CH_2_-CH(CH_3_)_2_), 1.39 (t, *J* = 6.9 Hz, 3H, -CH-CH_3_), 0.83 (dd, *J* = 11.5, 6.6 Hz, 6H, -CH_2_ CH(CH_3_)_2_); ^ 13^C-NMR (δ ppm): 175.48/170.27 (C_q_), 146.94/142.92 (N=CH), 139.95/139.20 (C_q_), 139.58 (C_q_), 134.71 (C_q_), 130.30/130.02 (CH_Ar_), 129.23 (CH_Ar_), 127.67 (CH_Ar_), 127.38 (CH_Ar_), 127.06 (CH_Ar_), 44.63 (-CH_2_-CH(CH_3_)_2_), 44.05 (-CH-CH_3_)/40.59 (-CH-CH_3_), 29.99 (-CH(CH_3_)_2_), 22.56 (-CH(CH_3_)_2_), 18.87 (-CH-CH_3_); HRMS (EI-MS): *m/z* calculated 309.196140; [M+H]^+^ found 309.196400; Green chemistry metrics: E-factor 16.001, ME 0.059.

*2-(4-Isobutylphenyl)propionic acid (4-chlorobenzylidene)hydrazide* (**3b**). 50/50 racemic mixture. ^1^H-NMR (δ ppm): 11.57/11.31 (s, 1H, -NH-N=), 8.20/7.91 (s, 1H, -N=CH), 7.79–7.59 (m, 2H, Ar-H), 7.46 (d, *J* = 8.4 Hz, 2H, Ar-H), 7.26 (t, *J* = 7.6 Hz, 2H, Ar-H), 7.07 (dd, *J* = 14.9, 7.6 Hz, 2H, Ar-H), 4.63/3.67 (q, *J* = 7.0 Hz, 1H, -CH-CH_3_), 2.37 (dd, *J* = 13.6, 6.9 Hz, 2H, -CH_2_-CH(CH_3_)_2_), 1.84–1.69 (m, 1H, -CH_2_-CH(CH_3_)_2_), 1.38 (t, *J* = 7.0 Hz, 3H, -CH-CH_3_), 0.81 (dd, *J* = 11.5, 6.9 Hz, 6H, -CH_2_-CH(CH_3_)_2_); ^13^C-NMR (δ ppm): 175.59/170.41 (C_q_), 145.68/141.68 (-N=CH-), 140.02/139.20 (C_q_), 139.62 (C_q_), 134.81/134.52 (C_q_), 133.73 (C_q_), 129.33 (2CH_Ar_), 129.02/128.74 (CH_Ar_), 127.72/127.47 (CH_Ar_), 44.69 (-CH_2_-CH(CH_3_)_2_, 44.11 (-CH-CH_3_)/40.59 (-CH-CH_3_), 29.99 (-CH-(CH_3_)_2_), 22.60 (-CH(CH_3_)_2_), 18.85 (-CH-CH_3_); HRMS (EI-MS): *m/z* calculated 343.157167; [M+H]^+^ found 343.157343; Green chemistry metrics: E-factor 11.942, ME 0.077.

*2-(4-Isobutylphenyl)propionic acid (4-bromobenzylidene)hydrazide* (**3c**). 50/50 racemic mixture. ^1^H-NMR (δ ppm): 11.57/11.32 (s, 1H, -NH-N=), 8.18/7.89 (s, 1H, -N=CH), 7.60 (d, *J* = 2.2 Hz, 4H, Ar-H), 7.26 (t, *J* = 7.6 Hz, 2H, Ar-H), 7.07 (dd, *J* = 15.1, 7.6 Hz, 2H, Ar-H), 4.63/3.67 (q, *J* = 6.9 Hz, 1H, -CH-CH_3_), 2.37 (dd, *J* = 13.0, 6.8 Hz, 2H, -CH_2_-CH-(CH_3_)_2_), 1.77 (dq, *J* = 20.3, 6.8 Hz, 1H, -CH_2_-CH-(CH_3_)_2_), 1.38 (t, *J* = 6.9 Hz, 3H, -CH-CH_3_), 0.81 (dd, *J* = 13.0, 6.8 Hz, 6H, -CH_2_-CH-(CH_3_)_2_); ^13^C-NMR (δ ppm): 175.11/169.93 (C_q_), 145.27/141.29 (-N=CH-), 139.53/138.70 (C_q_), 139.12 (C_q_), 133.59 (C_q_), 131.72 (CH_Ar_), 128.85 (CH_Ar_), 128.50 (CH_Ar_), 127.24/126.98 (CH_Ar_), 123.10/122.77 (C_q_), 44.21 (-CH_2_-CH(CH_3_)_2_), 43.64 (-CH-CH_3_)/40.18 (-CH-CH_3_), 29.56 (-CH-(CH_3_)_2_), 22.13 (-CH-(CH_3_)_2_), 18.43 (-CH-CH_3_); HRMS (EI-MS): *m/z* calculated 387.106652; [M+H]^+^ found 387.106804; Green chemistry metrics: E-factor 10.341, ME 0.088.

*2-(4-Isobutylphenyl)propionic acid (4-fluorobenzylidene)hydrazide* (**3d**). 50/50 racemic mixture. ^1^H-NMR (δ ppm): 11.50/11.25 (s, 1H, -NH-N=), 8.20/7.91 (s, 1H, -N=CH), 7.70 (dt, *J* = 9.0, 5.9 Hz, 2H, Ar-H), 7.27–7.23 (m, 4H, Ar-H), 7.08 (dd, *J* = 14.2, 9.0 Hz, 2H, Ar-H), 4.63/3.65 (q, *J* = 7.0 Hz, 1H, -CH-CH_3_), 2.38 (dd, *J* = 13.3, 6.7 Hz, 2H,-CH_2_-CH(CH_3_)_2_), 1.85–1.70 (m, 1H, -CH_2_-CH(CH_3_)_2_), 1.38 (t, *J* = 7.0 Hz, 3H, -CH-CH_3_), 0.82 (dd, *J* = 10.7, 6.7 Hz, 6H, -CH_2_**-**CH(CH_3_)_2_); ^ 13^C-NMR (δ ppm): 175.06/169.86 (***C_q_***), 164.14/161.04 (***C_q_***), 145.42/141.36 (-N=CH-), 139.54/138.78 (C_q_), 139.17 (C_q_), 131.32 (C_q_), 129.09 (CH_Ar_), 128.83 (CH_Ar_), 127.25/126.99 (CH_Ar_), 115.91/115.69 (CH_Ar_), 44.63 (-CH_2_-CH(CH_3_)_2_), 44.02/40.58 (-CH-CH_3_), 30.00 (-CH(CH_3_)_2_), 22.56 (**-**CH(CH_3_)_2_), 18.87 (-CH-CH_3_); HRMS (EI-MS): *m/z* calculated 327.186718; [M+H]^+^ found 327.186906; Green chemistry metrics: E-factor 13.705, ME 0.068.

*2-(4-Isobutylphenyl)propionic acid (2-nitrobenzylidene)hydrazide* (**3e**). 50/50 racemic mixture. ^1^H-NMR (δ ppm): 11.69 (s, 1H, -NH-N=), 8.61/8.30 (s, 1H, -N=CH), 8.01 (td, *J* = 14.3, 7.7 Hz, 2H, Ar-H), 7.76 (t, *J* = 7.7 Hz, 1H, Ar-H), 7.63 (q, *J* = 7.7 Hz, 1H, Ar-H), 7.31–7.20 (m, 2H, Ar-H), 7.09 (dd, *J* = 12.0, 8.6 Hz, 2H, Ar-H), 4.59/3.68 (q, *J* = 8.0 Hz, 1H, -CH-CH_3_), 2.44–2.33 (m, 2H, -CH_2_-CH(CH_3_)_2_), 1.79 (m, 1H, -CH_2_-CH(CH_3_)_2_), 1.45–1.32 (m, 3H, -CH-CH_3_), 0.82 (dd, *J* = 11.3, 7.2 Hz, 6H, -CH_2_-CH(CH_3_)_2_); ^13^C-NMR (δ ppm): 175.40/170.20 (C_q_), 148.01 (C_q_), 141.76/138.62 (-N=CH-), 139.63/139.27 (C_q_), 139.00/137.87 (C_q_), 133.54 (CH_Ar_), 130.34 (CH_Ar_), 128.94 (CH_Ar_), 128.75/128.40 (C_q_), 127.86 (CH_Ar_), 127.13 (CH_Ar_), 124.56 (CH_Ar_), 44.22 (-CH_2_-CH-(CH_3_)_2_), 43.71 (-CH-CH_3_)/40.24 (-CH-CH_3_), 29.58 (-CH(CH_3_)_2_), 22.14 (-CH(CH_3_)_2_), 18.52 (-CH-CH_3_); HRMS (EI-MS): *m/z* calculated 354.181218; [M+H]^+^ found 354.181363; Green chemistry metrics: E-factor 14.772, ME 0.063.

*2-(4-Isobutylphenyl)propionic acid (3-nitrobenzylidene)hydrazide* (**3f**). 50/50 racemic mixture. ^1^H-NMR (δ ppm): 11.75/11.48 (s, 1H, -NH-N=), 8.45 (d, *J* = 22.9 Hz, 1H, Ar-H), 8.31/8.00 (s, 1H, -N=CH), 8.20 (t, J = 8.0 Hz, 1H, Ar-H), 8.06 (dd, *J* = 11.2, 8.0 Hz, 1H, Ar-H), 7.78–7.60 (m, 1H, Ar-H), 7.27 (t, *J* = 7.5 Hz, 2H, Ar-H), 7.08 (dd, *J* = 12.9, 7.5 Hz, 2H, Ar-H), 4.60/3.70 (q, *J* = 6.9 Hz, 1H, -CH-CH_3_), 2.37 (dd, *J* = 17.0, 6.7 Hz, 2H, -CH_2_-CH-(CH_3_)_2_), 1.83–1.69 (m, 1H, -CH_2_-CH(CH_3_)_2_), 1.39 (dd, *J* = 9.1, 6.9 Hz, 3H, -CH-CH_3_), 0.80 (dd, J = 20.5, 6.7 Hz, 6H, -CH_2_-CH(CH_3_)_2_); ^13^C-NMR (δ ppm): 175.27/170.21 (C_q_), 148.16 (C_q_), 144.10/140.08 (-N=CH-), 139.60/139.20 (C_q_), 138.62 (C_q_), 136.20 (C_q_), 133.13/132.79 (CH_Ar_), 130.33 (CH_Ar_), 128.93 (CH_Ar_), 127.07 (CH_Ar_ ), 124.06/123.74 (CH_Ar_), 120.90/120.67 (CH_Ar_), 44.20 (-CH_2_-CH(CH_3_)_2_), 43.63/40.66 (-CH-CH_3_), 29.57 (-CH(CH_3_)_2_), 22.09 (-CH(CH_3_)_2_), 18.51 (-CH-CH_3_); HRMS (EI-MS): *m/z* calculated 354.181218; [M+H]^+^ found 354.181401; Green chemistry metrics: E-factor 14.123, ME 0.066.

*2-(4-Isobutylphenyl)propionic acid (4-nitrobenzylidene)hydrazide* (**3g**). 50/50 racemic mixture. ^1^H-NMR (δ ppm): 11.79/11.55 (s, 1H, -NH-N=), 8.30/8.00 (s, 1H, -N=CH), 8.25 (dd, *J* = 8.6, 3.5 Hz, 2H, Ar-H), 7.90 (dd, *J* = 8.6, 6.4 Hz, 2H, Ar-H), 7.27 (dd, *J* = 7.9, 1.8 Hz, 2H, Ar-H), 7.08 (dd, *J* = 13.2, 7.9 Hz, 2H, Ar-H), 4.65/3.70 (q, *J* = 6.9 Hz, 1H, -CH-CH_3_), 2.37 (dd, *J* = 15.1, 7.1 Hz, 2H, -CH_2_-CH(CH_3_)_2_), 1.88–1.66 (m, 1H, -CH_2_-CH(CH_3_)_2_), 1.39 (t, *J* = 6.9 Hz, 3H, -CH-CH_3_), 0.81 (dd, *J* = 14.2, 6.6 Hz, 6H, -CH_2_-CH(CH_3_)_2_); ^13^C-NMR (δ ppm): 175.44/170.27 (C_q_), 147.73/147.51 (C_q_), 144.07/140.64 (-N=CH-), 140.15 (C_q_), 139.63/139.28 (C_q_), 138.95/138.54 (C_q_), 128.94 (CH_Ar_), 127.84/127.55 (CH_Ar_), 127.24/127.01 (CH_Ar_), 123.97 (CH_Ar_), 44.20 (-CH_2_-CH(CH_3_)_2_), 43.70/40.35 (-CH-CH_3_), 29.56 (-CH(CH_3_)_2_), 22.12 (-CH(CH_3_)_2_), 18.44 (-CH-CH_3_); HRMS (EI-MS): *m/z* calculated 354.181218; [M+H]^+^ found 354.181362; Green chemistry metrics: E-factor 15.231, ME 0.062.

*2-(4-Isobutylphenyl)propionic acid (4-methylbenzylidene)hydrazide* (**3h**). 50/50 racemic mixture. Yield: 81%, m.p. 145–147 °C; IR (ZnSe crystal, cm^−1^): 3181 (-NH-), 2953 (CH_Ar_), 1666 (-CO-NH-), 1608 (-CH=N-); ^1^H-NMR (δ ppm): 11.44/11.18 (s, 1H, -NH-N=), 8.17/7.89 (s, 1H, -N=CH-), 7.54 (dd, *J* = 7.3, 5.2, 2H, Ar-H), 7.36–7.16 (m, 4H, Ar-H), 7.07 (dd, *J* = 15.5, 7.8 Hz, 2H, Ar-H), 4.64/3.66 (q, *J* = 6.8 Hz, 1H, -CH-CH_3_), 2.38 (dd, *J* = 13.8, 6.6 Hz, 2H, -CH_2_-CH(CH_3_)_2_), 2.31 (s, 3H, Ar-CH_3_), 1.77 (dd, *J* = 6.6, 3.6 Hz, 1H, -CH_2_-CH(CH_3_)_2_), 1.38 (t, *J* = 6.8 Hz, 3H, -CH-CH_3_), 0.82 (dd, *J* = 10.4, 6.6 Hz, 6H, -CH_2_-CH(CH_3_)_2_); ^13^C-NMR (δ ppm): 174.94/169.73 (C_q_), 146.54/142.56 (-N=CH-), 139.66/139.49 (C_q_), 139.33/138.83 (C_q_), 139.16 (C_q_), 131.60 (C_q_), 129.35 (CH_Ar_), 128.84 (CH_Ar_), 127.26/126.61 (CH_Ar_), 126.94 (CH_Ar_), 44.22 (-CH_2_-CH(CH_3_)_2_), 43.62/40.13 (-CH(CH_3_), 29.58 (-CH(CH_3_)_2_), 22.14 (-CH(CH_3_)_2_), 20.97 (Ar-CH_3_), 18.43 (-CH-CH_3_); HRMS (EI-MS): *m/z* calculated 323.211790; [M+H]^+^ found 323.212005; Green chemistry metrics: E-factor 14.262, ME 0.065.

*2-(4-Isobutylphenyl)propionic acid (3-trifluoromethylbenzylidene)hydrazide* (**3i**). 50/50 racemic mixture. Yield: 69%, m.p. 120–123 °C; IR (ZnSe crystal, cm^−1^): 3180 (-NH-), 2956 (CH_Ar_), 1668 (-CO-NH-), 1602 (-CH=N-), 1070 (C-F); ^1^H-NMR (δ ppm): 11.70/11.42 (s, 1H, -NH-N=), 8.28/7.99 (s, 1H, -N=CH-), 7.97–7.89 (m, 2 H, Ar-H), 7.74–7.61 (m, 2H, Ar-H), 7.25 (dd, *J* = 16.3, 7.6, 2H, Ar-H), 7.06 (dd, *J* = 19.3, 7.6 Hz, 2H, Ar-H), 4.58/3.69 (q, *J* = 6.9 Hz, 1H, -CH-CH_3_), 2.36 (dd, *J* = 17.9, 6.5 Hz, 2H, -CH_2_-CH(CH_3_)_2_), 1.85–1.66 (m, 1H, -CH_2_-CH(CH_3_)_2_), 1.38 (dd, *J* = 13.0, 6.9 Hz, 3H, ‑CH-CH_3_), 0.80 (dd, *J* = 20.8, 6.5 Hz, 6H, -CH_2_-CH(CH_3_)_2_); ^13^C-NMR (δ ppm): 175.27/170.21 (C_q_), 144.81/140.72 (-N=CH-), 139.63 (C_q_), 139.28 (C_q_), 138.69 (C_q_), 135.52 (C_q_), 130.87/130.55 (CH_Ar_), 129.91 (CH_Ar_), 128.93 (CH_Ar_), 127.08 (CH_Ar_), 126.12/125.78 (CH_Ar_), 125.38(C_q_), 122.98/122.70 (CH_Ar_), 44.24 (-CH_2_-CH(CH_3_)_2_), 43.65/40.78 (-CH-CH_3_), 29.60 (-CH(CH_3_)_2_), 22.11 (-CH(CH_3_)_2_), 18.54 (-CH-CH_3_); HRMS (EI-MS): *m/z* calculated 377.183524; [M+H]^+^ found 377.183680; Green chemistry metrics: E-factor 14.641, ME 0.064.

*2-(4-Isobutylphenyl)propionic acid (4-trifluoromethylbenzylidene)hydrazide* (**3j**). 50/50 racemic mixture. Yield: 69%, m.p. 160–162 °C; IR (ZnSe crystal, cm^−1^): 3185 (-NH-), 2957 (CH_Ar_), 1669 (-CO-NH-), 1604 (-CH=N-), 1064 (C-F); ^1^H-NMR (δ ppm): 11.69/11.45 (s, 1H, -NH-N=), 8.28/7.98 (s, 1H, -N=CH-), 7.86 (t, *J* = 8.2 Hz, 2H, Ar-H), 7.75 (dd, *J* = 8.2, 2.4 Hz, 2H, Ar-H), 7.27 (dd, *J* = 7.9, 6.2, 2H, Ar-H), 7.07 (dd, *J* = 15.6, 7.9 Hz, 2H, Ar-H), 4.65/3.69 (q, *J* = 6.9 Hz, 1H, -CH-CH_3_), 2.37 (dd, *J* = 16.0, 6.7 Hz, 2H, -CH_2_-CH(CH_3_)_2_), 1.76 (m, 1H, -CH_2_-CH(CH_3_)_2_), 1.39 (t, *J* = 6.9 Hz, 3H, ‑CH-CH_3_), 0.81 (dd, *J* = 16.6, 6.7 Hz, 6H, -CH_2_-CH(CH_3_)_2_); ^13^C-NMR (δ ppm): 175.31/170.15 (C_q_), 144.79/140.81 (-N=CH-), 139.60 (C_q_), 139.14 (C_q_), 138.64 (C_q_), 138.29 (C_q_), 128.91 (CH_Ar_), 127.50/127.01 (CH_Ar_), 127.22 (CH_Ar_), 125.60 (CH_Ar_), 122.73 (C_q_) 44.21 (-CH_2_-CH(CH_3_)_2_), 43.68/40.33 (-CH-CH_3_), 29.57 (-CH(CH_3_)_2_), 22.09 (-CH(CH_3_)_2_), 18.43 (-CH-CH_3_); HRMS (EI-MS): *m/z* calculated 377.183524; [M+H]^+^ found 377.183689; Green chemistry metrics: E-factor 14.822, ME 0.063.

*2-(4-Isobutylphenyl)propionic acid (4-cianobenzylidene)hydrazide* (**3k**). 50/50 racemic mixture. Yield: 52%, m.p. 179–180 °C; IR (ZnSe crystal, cm^−1^): 3180 (-NH-), 2954 (CH_Ar_), 2227 (C≡N), 1666 (-CO-NH-), 1596 (-CH=N-); ^1^H-NMR (δ ppm): 11.75/11.47 (s, 1H, -NH-N=), 8.25/7.95 (s, 1H, -N=CH-), 7.92–7.73 (m, 4H, Ar-H), 7.26 (t, *J* = 7.4, 2H, Ar-H), 7.07 (dd, *J* = 15.2, 7.4 Hz, 2H, Ar-H), 4.64/3.68 (q, *J* = 7.1 Hz, 1H, -CH-CH_3_), 2.37 (dd, *J* = 14.4, 6.8 Hz, 2H, -CH_2_-CH(CH_3_)_2_), 1.76 (dd, *J* = 12.5, 6.8 Hz, 1H, -CH_2_-CH(CH_3_)_2_), 1.38 (t, *J* = 7.1 Hz, 3H, -CH-CH_3_), 0.81 (dd, *J* = 13.8, 6.8 Hz, 6H, -CH_2_-CH(CH_3_)_2_); ^13^C-NMR (δ ppm): 175.45/170.29 (C_q_), 144.67/140.69 (-N=CH-), 139.68/139.32 (C_q_), 139.03/138.60 (C_q_), 138.80 (C_q_), 132.70 (CH_Ar_), 128.98 (CH_Ar_), 127.54/127.05 (CH_Ar_), 127.27 (CH_Ar_), 118.69 (C_q_), 111.79/111.51 (C_q_), 44.24 (-CH_2_-CH(CH_3_)_2_), 43.72/40.34 (-CH-CH_3_), 29.61 (-CH(CH_3_)_2_), 22.17 (-CH(CH_3_)_2_), 18.48 (-CH-CH_3_); HRMS (EI-MS): *m/z* calculated 334.191389; [M+H]^+^ found 334.191499; Green chemistry metrics: E-factor 22.513, ME 0.042.

*2-(4-Isobutylphenyl)propionic acid (2,6-dichlorobenzylidene)hydrazide* (**3l**). 60/40 racemic mixture. Yield: 60%, m.p. 151–153 °C; IR (ZnSe crystal, cm^−1^): 3185 (-NH-), 2951 (CH_Ar_), 1662 (-CO-NH-), 1605 (-CH=N-), 776 (C-Cl); ^1^H-NMR (δ ppm): 11.77/11.52 (s, 1H, -NH-N=), 8.41/8.22 (s, 1H, -N=CH-), 7.51 (d, *J* = 7.9 Hz, 2H, Ar-H), 7.43–7.34 (m, 1H, Ar-H), 7.24 (dd, *J* =29.5, 8.0 Hz, 2H, Ar-H), 7.07 (dd, *J* = 29.5, 8.0, 2H, Ar-H), 4.59/3.67 (q, *J* = 7.0 Hz, 1H, -CH-CH_3_), 2.37 (dd, *J* = 14.8, 6.8 Hz, 2H, -CH_2_-CH(CH_3_)_2_), 1.86–1.67 (m, 1H, -CH_2_-CH(CH_3_)_2_), 1.38 (dd, *J* = 15.8, 7.0 Hz, 3H, -CH-CH_3_), 0.82 (dd, *J* = 10.2, 6.8 Hz, 6H, -CH_2_-CH(CH_3_)_2_); ^13^C-NMR (δ ppm): 175.55/170.12 (C_q_), 141.97/137.97 (-N=CH-), 139.70/139.29 (C_q_), 138.76/138.61 (C_q_), 133.88 (C_q_), 131.07/130.87 (CH_Ar_), 130.47 (C_q_), 129.74 (C_q_), 129.34 (CH_Ar_), 128.93 (CH_Ar_), 127.20 (CH_Ar_), 44.26 (-CH_2_-CH(CH_3_)_2_), 43.78/39.85 (-CH-CH_3_), 29.62 (-CH(CH_3_)_2_), 22.16 (-CH(CH_3_)_2_), 18.64 (-CH-CH_3_); HRMS (EI-MS): *m/z* calculated 377.118195; [M+H]^+^ found 377.118261; Green chemistry metrics: E-factor 16.891, ME 0.056.

#### 3.2.2. Preparation of the Acetamidothiazolidin-4-One Derivatives **4a**–**l**

The acyl hydrazones of ibuprofen **3a**–**l** (3.2 mmol) were reacted with mercaptoacetic acid (14.4 mmol) and the reaction mixtures was heated at 80–85 °C for 2–8 h using an oil bath, according to a procedure described for other compounds [32]. After the completion of the reaction (TLC monitoring using dichloromethane-methanol, 9.8:0.2, v/v, UV light at 254 nm), the mixture was diluted with ethyl acetate (200 mL) and then neutralized with a saturated solution of sodium carbonate. After stirring for 3–4 h the organic layer was separated and washed with saturated solution of sodium bicarbonate, then with saturated solution of sodium chloride and finally with water. The organic layers were collected, dried with MgSO_4_ and concentrated by rotary evaporator. The residue was purified on a silica gel column using dichloromethane-methanol (9.8:0.2, v/v) as the eluent system.

*2-(Phenyl)-3-[2-(4-(isobutyl)phenyl)-2-methyl]acetamidothiazolidine-4-one* (**4a**). 60/40 racemic mixture. Yield: 57%, m.p. 125 °C; IR (ZnSe crystal, cm^−1^): 3243 (-NH-), 2959 (CH_Ar_), 1716 (C=O, thiazolidine-4-one), 1666 (-CO-NH-), 1203 (C-N), 694 (C-S); ^1^H-NMR (δ ppm): 10.26 (d, *J* = 3.1 Hz, 1H, -NH-), 7.50–7.35 (m, 2H, Ar-H), 7.34–7.21 (m, 3H, Ar-H), 7.11 (d, *J* = 7.6 Hz, 1H, Ar-H), 7.01 (dd, *J* = 14.8, 7.6 Hz, 3H, Ar-H), 5.82/5.67 (s, 1H, CH thiazolidine-4-one), 3.86 (t, *J* = 14.9 Hz, 1H, CH_2_ thiazolidine-4-one), 3.72 (dd, *J* = 14.9, 9.0 Hz, 1H, CH_2_ thiazolidine-4-one), 3.61–3.47 (m, 1H, -CH-CH_3_), 2.39 (d, *J* = 6.7 Hz, 2H, -CH_2_-CH(CH_3_)_2_), 1.79 (dt, *J* = 13.4, 6.7 Hz, 1H, -CH_2_-CH(CH_3_)_2_), 1.25 (dd, *J* = 24.2, 7.0 Hz, 3H, -CH-CH_3_), 0.85 (d, *J* = 6.7 Hz, 6H, -CH_2_-CH(CH_3_)_2_); ^13^C-NMR (δ ppm): 172.23 (C_q_), 168.85/168.69 (C_q_), 139.31 (C_q_), 138.15 (C_q_), 137.95 (C_q_), 128.98/128.88 (CH_Ar_), 128.69 (CH_Ar_), 128.54/128.39 (CH_Ar_), 127.80 (CH_Ar_), 127.01/126.73 (CH_Ar_), 61.43 (CH thiazolidine-4-one), 44.22 (-CH_2_-CH(CH_3_)_2_), 42.12 (-CH-CH_3_), 29.62 (-CH(CH_3_)_2_), 29.27 (CH_2_ thiazolidine-4-one), 22.13 (-CH(CH_3_)_2_), 18.81/17.90 (-CH-CH_3_); HRMS (EI-MS): *m/z* calculated 383.178776; [M+H]^+^ found 383.178716; Green chemistry metrics: E-factor 2.361, ME 0.297.

*2-(4-Chlorophenyl)-3-[2-(4-(isobutyl)phenyl)-2-methyl]acetamidothiazolidine-4-one* (**4b**). 50/50 racemic mixture. Yield: 76%, m.p. 80 °C; IR (ZnSe crystal, cm^−1^): 3238 (-NH-), 2954 (CH_Ar_), 1718 (C=O, thiazolidine-4-one), 1672 (-CO-NH-), 1215 (C-N), 810 (C-Cl), 656 (C-S); ^1^H-NMR (δ ppm): 10.23 (d, *J* = 7.1 Hz, 1H, -NH-), 7.43 (q, *J* = 8.6 Hz, 2H, Ar-H), 7.28 (dd, *J* = 18.7, 8.6 Hz, 2H, Ar-H), 7.16–6.88 (m, 4H, Ar-H), 5.80/5.71 (s, 1H, CH thiazolidine-4-one), 3.87 (dd, *J* = 15.8, 8.3, 1H, CH_2_ thiazolidine-4-one), 3.72 (dd, *J* = 15.8, 8.3 Hz, 1H, CH_2_ thiazolidine-4-one), 3.52 (dq, *J* = 14.1, 7.0 Hz, 1H, -CH-CH_3_), 2.46–2.33 (m, 2H, -CH_2_-CH(CH_3_)_2_), 1.79 (dt, *J* = 13.5, 6.7 Hz, 1H, -CH_2_-CH(CH_3_)_2_), 1.25 (dd, *J* = 17.9, 7.0 Hz, 3H, -CH-CH_3_), 0.85 (d, *J* = 6.7 Hz, 6H, -CH_2_-CH(CH_3_)_2_); ^13^C-NMR (δ ppm): 172.17 (C_q_), 168.70/168.45 (C_q_), 139.36 (C_q_), 138.17/137.94 (C_q_), 137.25/137.07 (C_q_), 133.56/133.44 (C_q_), 129.77 (CH_Ar_), 128.70 (CH_Ar_), 128.49/128.38 (CH_Ar_), 126.92/126.71 (CH_Ar_), 60.86/60.50 (CH thiazolidine-4-one), 44.22 (-CH_2_-CH(CH_3_)_2_), 42.21 (-CH-CH_3_), 29.61 (-CH(CH_3_)_2_), 29.21 (CH_2_ thiazolidine-4-one), 22.15 (-CH(CH_3_)_2_), 18.76/17.89 (-CH-CH_3_); HRMS (EI-MS): *m/z* calculated 417.139803; [M+H]^+^ found 417.139745; Green chemistry metrics: E-factor 1.423, ME 0.413.

*2-(4-Bromophenyl)-3-[2-(4-(isobutyl)phenyl)-2-methyl]acetamidothiazolidine-4-one* (**4c**). 50/50 racemic mixture. Yield: 77%, m.p. 70–72 °C; IR (ZnSe crystal, cm^−1^): 3238 (-NH), 2954 (CH_Ar_), 1718 (C=O, thiazolidine-4-one), 1672 (-CO-NH-), 1215 (C-N), 810 (C-Cl), 656 (C-S); ^1^H-NMR (δ ppm): 10.23 (d, *J* = 8.1 Hz, 1H, -NH-), 7.55 (d, *J* = 8.4 Hz, 1H, Ar-H), 7.41 (dd, *J* = 21.1, 8.4 Hz, 2H, Ar-H), 7.19 (d, *J* = 8.4 Hz, 1H, Ar-H), 7.11–6.93 (m, 4H, Ar-H), 5.79/5.70 (s, 1H, CH thiazolidine-4-one), 3.87 (dd, *J* = 15.8, 8.4 Hz, 1H, CH_2_ thiazolidine-4-one), 3.72 (dd, *J* = 15.8, 8.4 Hz, 1H, CH_2_ thiazolidine-4-one), 3.52 (dq, *J* = 14.1, 7.0 Hz, 1H, -CH-CH_3_), 2.40 (dd, *J* = 6.9, 2.3 Hz, 2H, -CH_2_-CH(CH_3_)_2_), 1.79 (td, *J* = 13.4, 6.9 Hz, 1H, -CH_2_-CH(CH_3_)_2_), 1.25 (dd, *J* = 18.2, 7.0 Hz, 3H, -CH-CH_3_), 0.85 (d, *J* = 6.9 Hz, 6H, -CH_2_-CH(CH_3_)_2_); ^13^C-NMR (δ ppm): 172.16 (C_q_), 168.71/168.46 (C_q_), 139.35 (C_q_), 138.16/137.95 (C_q_), 137.68/137.48 (C_q_), 131.36 (CH_Ar_), 130.06 (CH_Ar_), 128.70 (CH_Ar_), 126.91/126.71 (CH_Ar_), 122.22/122/08 (C_q_), 60.94/60.56 (CH thiazolidine-4-one), 44.24 (-CH_2_-CH(CH_3_)_2_), 42.21 (-CH-CH_3_), 29.62 (-CH(CH_3_)_2_), 29.22 (CH_2_ thiazolidine-4-one), 22.19 (-CH(CH_3_)_2_), 18.77/17.89 (-CH-CH_3_); HRMS (EI-MS): *m/z* calculated 461.089288; [M+H]^+^ found 461.089204; Green chemistry metrics: E-factor 1.122, ME 0.471. 

*2-(4-Fluorophenyl)-3-[2-(4-(isobutyl)phenyl)-2-methyl]acetamidothiazolidine-4-one* (**4d**). 50/50 racemic mixture. Yield: 66%, m.p. 72 °C; IR (ZnSe crystal, cm^−1^): 3252 (-NH-), 2954 (CH_Ar_), 1716 (C=O, thiazolidine-4-one), 1672 (-CO-NH-), 1224 (C-N), 1095 (C-F), 659 (C-S); ^1^H-NMR (δ ppm): 10.22 (d, *J* = 4.4 Hz, 1H, -NH-), 7.48 (dd, *J* = 8.8, 5.5 Hz, 1H, Ar-H), 7.28 (dd, *J* = 8.6, 5.5 Hz, 1H, Ar-H), 7.18 (t, *J* = 8.8 Hz, 1H, Ar-H), 7.14–6.90 (m, 5H, Ar-H), 5.82/5.71 (s, 1H, CH thiazolidine-4-one), 3.86 (dd, *J* = 15.8, 8.6, 1.4 Hz, 1H, CH_2_ thiazolidine-4-one), 3.72 (dd, *J* = 15.8, 8.6 Hz, 1H, CH_2_ thiazolidine-4-one), 3.52 (dq, *J* = 14.1, 7.0 Hz, 1H, -CH-CH_3_), 2.39 (dd, *J* = 6.7, 1.2 Hz, 2H, -CH_2_-CH(CH_3_)_2_), 1.79 (dt, *J* = 13.5, 6.7 Hz, 1H, -CH_2_-CH(CH_3_)_2_), 1.25 (dd, *J* = 17.2, 7.0 Hz, 3H, -CH-CH_3_), 0.85 (d, *J* = 6.7 Hz, 6H, -CH_2_-CH(CH_3_)_2_); ^13^C-NMR (δ ppm): 172.18 (C_q_), 168.70/168.47 (C_q_), 163.54/161.10 (C_q_), 139.35 (C_q_), 138.19/137.95 (C_q_), 134.21(C_q_), 130.16 (CH_Ar_), 128.70 (CH_Ar_), 126.85 (CH_Ar_), 115.27 (CH_Ar_), 60.90/60.59 (CH thiazolidine-4-one), 44.22 (-CH_2_-CH(CH_3_)_2_), 42.21 (-CH-CH_3_), 29.62 (-CH(CH_3_)_2_), 29.26 (CH_2_ thiazolidine-4-one), 22.15 (CH(CH_3_)_2_), 18.76/17.89 (-CH-CH_3_); HRMS (EI-MS): *m/z* calculated 401.169354; [M+H]^+^ found 401.169387; Green chemistry metrics: E-factor 1.821, ME 0.354.

*2-(2-Nitrophenyl)-3-[2-(4-(isobutyl)phenyl)-2-methyl]acetamidothiazolidine-4-one* (**4e**)*.* 70/30 racemic mixture. Yield: 73%, m.p. 69–71 °C; IR (ZnSe crystal, cm^−1^): 3246 (-NH), 2953 (CH_Ar_), 1721 (C=O, thiazolidine-4-one), 1674 (-CO-NH-), 1216 (C-N), 661 (C-S); ^1^H-NMR (δ ppm): 10.45/10.38 (d, 1H, -NH-), 8.05/7.96 (d, *J* = 8.2 Hz, 1H, Ar-H), 7.90–7.49 (m, 3H, Ar-H), 7.12/7.03 (d, *J* = 8.1 Hz, 3H, Ar-H), 6.90 (d, *J* = 6.5 Hz, 1H, Ar-H), 6.14/6.02 (d, *J* = 1.4 Hz, 1H, CH thiazolidine-4-one), 3.93–3.86 (m, 1H, CH_2_ thiazolidine-4-one), 3.70 (dd, *J* = 20.0, 15.8, 1H, CH_2_ thiazolidine-4-one), 3.57–3.48 (m, 1H, -CH-CH_3_), 2.37 (dd, *J* = 20.3, 6.8 Hz, 2H, -CH_2_-CH(CH_3_)_2_), 1.84–1.69 (m, 1H, -CH_2_-CH(CH_3_)_2_), 1.24 (dd, *J* = 23.1, 7.0 Hz, 3H, -CH-CH_3_), 0.83 (dd, *J* = 13.4, 6.8 Hz, 6H, -CH_2_-CH(CH_3_)_2_); ^13^C-NMR (δ ppm): 172.38 (C_q_), 169.07/168.89 (C_q_), 147.67/147.45 (C_q_), 139.38/139.31 (C_q_), 138.01/137.92 (C_q_), 134.55 (CH_Ar_), 134.33 (C_q_), 129.75 (CH_Ar_), 128.72 (CH_Ar_), 128.16/127.94 (CH_Ar_), 126.93 (CH_Ar_), 126.35 (CH_Ar_), 124.79 (CH_Ar_), 56.98/56.48 (CH thiazolidine-4-one), 44.19 (-CH_2_-CH(CH_3_)_2_), 42.26 (-CH-CH_3_), 29.57 (-CH(CH_3_)_2_), 28.45 (-CH_2_ thiazolidine-4-one), 22.14 (-CH(CH_3_)_2_), 18.68/17.83 (-CH-CH_3_); HRMS (EI-MS): *m/z* calculated 428.163854; [M+H]^+^ found 428.163864; Green chemistry metrics: E-factor 1.503, ME 0.405.

*2-(3-Nitrophenyl)-3-[2-(4-(isobutyl)phenyl)-2-methyl]acetamidothiazolidine-4-one* (**4f**). 50/50 racemic mixture. Yield: 62%, m.p. 70–72 °C; IR (ZnSe crystal, cm^−1^): 3247 (-NH-), 2953 (CH_Ar_), 1721 (C=O, thiazolidine-4-one), 1674 (-CO-NH-), 1216 (C-N), 679 (C-S); ^1^H-NMR (δ ppm): 10.31/10.25 (d, 1H, -NH-), 8.37–8.04 (m, 2H, Ar-H), 7.91/7.72 (d, *J* = 7.7 Hz, 1H, Ar-H), 7.60 (dt, *J* = 29.4, 7.7 Hz, 1H, Ar-H), 7.16–6.76 (m, 4H, Ar-H), 5.96/5.90 (s, 1H, CH thiazolidine-4-one), 3.97–3.92 (m, 1H, CH_2_ thiazolidine-4-one), 3.77 (dd, *J* = 15.8, 8.3, 1H, CH_2_ thiazolidine-4-one), 3.49 (dq, *J* = 14.1, 6.9 Hz, 1H, -CH-CH_3_), 2.35 (t, *J* = 7.5 Hz, 2H, -CH_2_-CH(CH_3_)_2_), 1.83–1.67 (m, 1H, -CH_2_-CH(CH_3_)_2_), 1.23 (dd, *J* = 20.2, 6.9 Hz, 3H, -CH-CH_3_), 0.81 (t, *J* = 7.1 Hz, 6H, -CH_2_-CH(CH_3_)_2_); ^13^C-NMR (δ ppm): 172.16 (C_q_), 168.71/168.39 (C_q_), 147.66 (C_q_), 140.70 (C_q_), 139.37 (C_q_), 138.01 (C_q_), 134.80/134.55 (CH_Ar_), 130.08 (CH_Ar_), 128.65 (CH_Ar_), 126.71 (CH_Ar_), 123.82/122.69 (CH_Ar_), 60.66/60.20 (CH thiazolidine-4-one), 44.17 (-CH_2_-CH(CH_3_)_2_), 42.29 (-CH-CH_3_), 29.56 (-CH(CH_3_)_2_), 29.21 (CH_2_ thiazolidine-4-one), 22.15 (-CH(CH_3_)_2_), 18.70/17.76 (-CH-CH_3_); HRMS (EI-MS): *m/z* calculated 428.163854; [M+H]^+^ found 428.163708; Green chemistry metrics: E-factor 1.874, ME 0.352.

*2-(4-Nitrophenyl)-3-[2-(4-(isobutyl)phenyl)-2-methyl]acetamidothiazolidine-4-one* (**4g**). 50/50 racemic mixture. Yield: 72%, m.p. 70–72 °C; IR (ZnSe crystal, cm^−1^): 3233 (-NH-), 2955 (CH_Ar_), 1716 (C=O, thiazolidine-4-one), 1672 (-CO-NH-), 1217 (C-N), 692 (C-S); ^1^H-NMR (δ ppm): 10.31 (d, *J* = 11.5 Hz, 1H, -NH-), 8.17 (d, *J* = 8.4 Hz, 1H, Ar-H), 8.09 (d, *J* = 8.6 Hz, 1H, Ar-H), 7.70 (d, *J* = 8.4 Hz, 1H, Ar-H), 7.53 (d, *J* = 8.6 Hz, 1H, Ar-H), 7.16–6.82 (m, 4H, Ar-H), 5.93/5.89 (s, 1H, CH thiazolidine-4-one), 3.93 (d, *J* = 15.8, 1H, CH_2_ thiazolidine-4-one), 3.76 (dd, *J* = 15.8, 10.4, 1H, CH_2_ thiazolidine-4-one), 3.60–3.42 (m, 1H, -CH-CH_3_), 2.37 (d, *J* = 7.0 Hz, 2H, -CH_2_-CH(CH_3_)_2_), 1.82–1.69 (m, 1H, -CH_2_-CH(CH_3_)_2_), 1.24 (dd, *J* = 17.2, 6.9 Hz, 3H, -CH-CH_3_), 0.82 (t, *J* = 7.0 Hz, 6H, ‑CH_2_-CH(CH_3_)_2_); ^13^C-NMR (δ ppm): 172.20 (C_q_), 168.74/168.44 (C_q_), 147.64/147.50 (C_q_), 145.88 (C_q_), 139.38 (C_q_), 138.19/137.91 (C_q_), 129.06 (CH_Ar_), 128.66 (CH_Ar_), 126.77 (CH_Ar_), 123.53 (CH_Ar_), 60.55/60.01 (CH thiazolidine-4-one), 44.15 (-CH_2_-CH(CH_3_)_2_), 42.28 (-CH-CH_3_), 29.58 (-CH(CH_3_)_2_), 29.14 (CH_2_ thiazolidine-4-one), 22.13 (-CH(CH_3_)_2_), 18.60/17.91 (-CH-CH_3_); HRMS (EI-MS): *m/z* calculated 428.163854; [M+H]^+^ found 428.163805; Green chemistry metrics: E-factor 1.506, ME 0.403.

*2-(4-Methylphenyl)-3*-[2-(4-(isobutyl)phenyl)-2-methyl]*acetamidothiazolidine-4-one* (**4h**). 50/50 racemic mixture. Yield: 41%, m.p. 67 °C; IR (ZnSe crystal, cm^−1^): 3254 (-NH-), 2953 (CH_Ar_), 1715 (C=O thiazolidine-4-one), 1673 (-CO-NH-), 1213 (C-N), 659 (C-S); ^1^H-NMR (δ ppm): 10.21 (d, *J* = 4.0 Hz, 1H, -NH-), 7.31 (d, *J* = 7.9 Hz, 1H, Ar-H), 7.19 (d, *J* = 7.9 Hz, 1H, Ar-H), 7.16–7.05 (m, 3H, Ar-H), 7.05–6.96 (m, 3H, Ar-H), 5.78/5.64 (s, 1H, CH thiazolidine-4-one), 3.92–3.77 (m, 1H, CH_2_ thiazolidine-4-one), 3.71 (dd, *J* = 15.8, 9.0, 1H, CH_2_ thiazolidine-4-one), 3.53 (dq, *J* = 20.4, 7.0 Hz, 1H, -CH-CH_3_), 2.40 (dd, *J* = 7.0, 2.0 Hz, 2H, -CH_2_-CH(CH_3_)_2_), 2.30 (d, *J* = 21.4 Hz, 3H, Ar-CH_3_), 1.85–1.75 (m, 1H, -CH_2_-CH(CH_3_)_2_), 1.25 (dd, *J* = 20.4, 7.0 Hz, 3H, -CH-CH_3_), 0.85 (dd, *J* = 7.0, 2.9 Hz, 6H, -CH_2_-CH(CH_3_)_2_); ^13^C-NMR (δ ppm): 172.19 (C_q_), 168.82/168.63 (C_q_), 139.30 (C_q_), 138.46/138.31 (C_q_), 138.19.137.93 (C_q_), 135.05/134.88 (C_q_), 129.02 (CH_Ar_), 128.68 (CH_Ar_), 127.82 (CH_Ar_), 127.02/126.75 (CH_Ar_), 61.39/61.21 (CH thiazolidine-4-one), 44.22 (-CH_2_-CH(CH_3_)_2_), 42.11 (-CH-CH_3_), 29.63 (-CH(CH_3_)_2_), 29.28 (CH_2_ thiazolidine-4-one), 22.13 (-CH(CH_3_)_2_), 20.77 (Ar-CH_3_), 18.81/17.99 (-CH-CH_3_); HRMS (EI-MS): *m/z* calculated 397.194426; [M+H]^+^ found 397.194552; Green chemistry metrics: E-factor 3.767, ME 0.213.

*2-(3-trifluoromethylphenyl)-3-[2-(4-(isobutyl)phenyl)-2-methyl]acetamidothiazolidine-4-one* (**4i**). 50/50 racemic mixture. Yield: 53%, m.p. 68 °C; IR (ZnSe crystal, cm^−1^): 3233 (-NH-), 2955 (CH_Ar_), 1720 (C=O, thiazolidine-4-one), 1673 (-CO-NH-), 1216 (C-N), 1071 (C-F), 656 (C-S); ^1^H-NMR (δ ppm): 10.32 (d, *J* = 19.4 Hz, 1H, -NH-), 7.88–7.46 (m, 4H, Ar-H), 7.20–6.86 (m, 4H, Ar-H), 5.96/5.86 (s, 1H, CH thiazolidine-4-one), 3.96–3.90 (m, 1H, CH_2_ thiazolidine-4-one), 3.76 (dd, *J* = 15.8, 8.2, 1H, CH_2_ thiazolidine-4-one), 3.60–3.48 (m, 1H, -CH-CH_3_), 2.37 (dd, *J* = 6.8, 3.5 Hz, 2H, -CH_2_-CH(CH_3_)_2_), 1.85–1.70 (m, 1H, -CH_2_-CH(CH_3_)_2_), 1.26 (dd, *J* = 18.7, 7.0 Hz, 3H, -CH-CH_3_), 0.83 (dd, *J* = 6.8, 3.5 Hz, 6H, -CH_2_-CH(CH_3_)_2_); ^13^C-NMR (δ ppm): 172.33 (C_q_), 168.88/.16862 (C_q_), 139.86 (C_q_), 139.38 (C_q_), 138.07 (C_q_), 132.06 (CH_Ar_), 129.61 (CH_Ar_), 128.71 (CH_Ar_), 126.92/126.68 (CH_Ar_), 125.72/124.55 (CH_Ar_), 125.36 (C_q_), 122.66 (C_q_), 61.01/60.77 (CH thiazolidine-4-one), 44.29 (-CH_2_-CH(CH_3_)_2_, 42.33 (-CH-CH_3_), 29.62 (-CH(CH_3_)_2_), 29.32 (CH_2_ thiazolidine-4-one), 22.13 (-CH(CH_3_)_2_), 18.80/17.87 (-CH-CH_3_); HRMS (EI-MS): *m/z* calculated 451.166160; [M+H]^+^ found 451.166296; Green chemistry metrics: E-factor 2.079, ME 0.325.

*2-(4-Trifluoromethylphenyl)-3-[2-(4-(isobutyl)phenyl)-2-methyl]acetamidothiazolidine-4-one* (**4j**). 50/50 racemic mixture. Yield: 38%, m.p. 74–75 °C; IR (ZnSe crystal, cm^−1^): 3246 (-NH-), 2956 (CH_Ar_), 1721 (C=O, thiazolidine-4-one), 1673 (-CO-NH-), 1217 (C-N), 1066 (C-F), 664 (C-S); ^1^H-NMR (δ ppm): 10.28 (d, *J* = 9.6 Hz, 1H, -NH-), 7.77–7.54 (m, 3H, Ar-H), 7.48 (d, *J* = 8.1 Hz, 1H, Ar-H), 7.14‒6.89 (m, 4H, Ar-H), 5.89/5.82 (s, 1H, CH thiazolidine-4-one), 3.91 (dd, *J* = 15.7, 5.5 Hz, 1H, CH_2_ thiazolidine-4-one), 3.75 (dd, *J* = 15.7, 10.6, 1H, CH_2_ thiazolidine-4-one), 3.53 (dq, *J* =13.9, 7.0 Hz, 1H, -CH-CH_3_), 2.38 (d, *J* = 6.8 Hz, 2H, -CH_2_-CH(CH_3_)_2_), 1.85–1.69 (m, 1H, -CH_2_-CH(CH_3_)_2_), 1.25 (dd, *J* = 17.5, 7.0 Hz, 3H, -CH-CH_3_), 0.83 (dd, *J* = 6.8, 3.9 Hz, 6H, -CH_2_-CH(CH_3_)_2_); ^13^C-NMR (δ ppm): 172.22 (C_q_), 168.81/168.56 (C_q_), 143.19 (C_q_), 142.95 (C_q_), 139.34 (C_q_), 138.21 (C_q_), 137.97 (C_q_), 128.66 (CH_Ar_), 126.90 (CH_Ar_), 126.69 (CH_Ar_), 125.35 (CH_Ar_), 60.86/60.41 (CH thiazolidine-4-one), 44.20 (-CH_2_-CH(CH_3_)_2_), 42.24 (-CH-CH_3_), 29.58 (-CH(CH_3_)_2_), 29.19 (CH_2_ thiazolidine-4-one), 22.13 (-CH(CH_3_)_2_), 18.72/17.88 (-CH-CH_3_); HRMS (EI-MS): *m/z* calculated 451.166160; [M+H]^+^ found 451.166066; Green chemistry metrics: E-factor 3.687, ME 0.2134.

*2-(4-Cyanophenyl)-3-[2-(4-(isobutyl)phenyl)-2-methyl]acetamidothiazolidine-4-one* (**4k**). 50/50 racemic mixture. Yield: 87%, m.p. 75 °C; IR (ZnSe crystal, cm^−1^): 3256 (-NH-), 2953 (CH_Ar_), 2230 (C≡N), 1720 (C=O, thiazolidine-4-one), 1677 (-CO-NH-), 1204 (C-N), 661 (C-S); ^1^H-NMR (δ ppm): 10.28 (d, *J* = 10.7 Hz, 1H, -NH-), 7.81 (d, *J* = 8.0 Hz, 1H, Ar-H), 7.72 (d, *J* = 8.2 Hz, 1H, Ar-H), 7.61 (d, *J* = 8.2 Hz, 1H, Ar-H), 7.45 (d, *J* = 8.0 Hz, 1H, Ar-H), 7.11–6.90 (m, 4H, Ar-H), 5.87/5.81 (s, 1H, CH thiazolidine-4-one), 3.91 (dd, *J* = 15.8, 4.4 Hz, 1H, CH_2_ thiazolidine-4-one), 3.73 (dd, *J* = 15.8, 10.1, 1H, CH_2_ thiazolidine-4-one), 3.50 (dq, *J* = 14.1, 7.0 Hz, 1H, -CH-CH_3_), 2.39 (dd, *J* = 6.8, 2.5 Hz, 2H, -CH_2_-CH(CH_3_)_2_), 1.84–1.71 (m, 1H, -CH_2_-CH(CH_3_)_2_), 1.24 (dd, *J* = 16.6, 7.0 Hz, 3H, -CH-CH_3_), 0.83 (dd, J = 6.8, 2.5 Hz, 6H, -CH_2_-CH(CH_3_)_2_); ^13^C-NMR (δ ppm): 172.23 (C_q_), 168.77/168.49 (C_q_), 144.02/143.87 (C_q_), 139.40 (C_q_), 138.15/137.92 (C_q_), 132.41 (CH_Ar_), 128.69 (CH_Ar_), 126.90 (CH_Ar_), 126.68 (CH_Ar_), 118.48 (C_q_), 111.52 (C_q_), 60.85/60.39 (CH thiazolidine-4-one), 44.20 (-CH_2_-CH(CH_3_)_2_), 42.27 (-CH-CH_3_), 29.62 (-CH(CH_3_)_2_), 29.14 (CH_2_ thiazolidine-4-one), 22.15 (-CH(CH_3_)_2_), 18.68/17.91 (-CH-CH_3_); HRMS (EI-MS): *m/z* calculated 408.174025; [M+H]^+^ found 408.173935; Green chemistry metrics: E-factor 1.218, ME 0.452.

*2-(2,6-Dichlorophenyl)-3-[2-(4-(isobutyl)phenyl)-2-methyl]acetamidothiazolidine-4-one* (**4l**). 60/40 racemic mixture. Yield: 38%, m.p. 74 °C; IR (ZnSe crystal, cm^−1^): 3239 (-NH), 2953 (CH_Ar_), 1716 (C=O, thiazolidine-4-one), 1673 (-CO-NH-), 1218 (C-N), 778 (C-Cl), 663 (C-S); ^1^H-NMR (δ ppm): 10.38 (d, *J* = 10.9 Hz, 1H, -NH-), 7.61–7.24 (m, 3H, Ar-H), 7.22–6.89 (m, 4H, Ar-H), 6.71/6.57 (s, 1H, CH thiazolidine-4-one), 3.96–3.71 (m, 2H, CH_2_ thiazolidine-4-one), 3.68–3.50 (m, 1H, -CH-CH_3_), 2.38 (dd, *J* = 10.5, 6.5 Hz, 2H, -CH_2_-CH(CH_3_)_2_), 1.88–1.67 (m, 1H, -CH_2_-CH(CH_3_)_2_), 1.26 (dd, *J* = 22.0, 6.9 Hz, 3H, -CH-CH_3_), 0.84 (dd, *J* = 6.5, 2.8 Hz, 6H, -CH_2_-CH(CH_3_)_2_); ^13^C-NMR (δ ppm): 172.84/172.68 (C_q_), 168.19/168.01 (C_q_), 139.48/138.25 (C_q_), 138.06/137.74 (C_q_), 135.58/135.48 (C_q_), 135.25/134.72 (C_q_), 130.96 (C_q_), 131.38 (CH_Ar_), 131.14 (CH_Ar_), 130.89 (CH_Ar_), 128.68 (CH_Ar_), 127.08/126.46 (CH_Ar_), 55.87/55.72 (CH thiazolidine-4-one), 44.23 (-CH_2_-CH(CH_3_)_2_), 41.90 (-CH-CH_3_), 30.27 (-CH(CH_3_)_2_), 29.64 (CH_2_ thiazolidine-4-one), 22.18 (-CH(CH_3_)_2_), 18.84/17.49 (-CH-CH_3_); HRMS (EI-MS): *m/z* calculated 451.100831; [M+H]^+^ found 451.100760; Green chemistry metrics: E-factor 3.418, ME 0.226. 

#### 3.2.3. Preparation of 2-(4-Aminophenyl)-3-[2-(4-(isobutyl)phenyl)-2-methyl]acetamidothiazolidine-4-one (**4m**)

To a solution of 2-(4-nitrophenyl)-3-[2-(4-(isobutyl)phenyl)-2-methyl]acetamidothiazolidine-4-one (**4g**, 1.6 mmol) in ethyl alcohol (20 mL) there were added SnCl_2_ (10 mmol) and 3–4 drops of acetic acid and the mixture of reaction was stirred at room temperature under argon atmosphere for 14 h, according to the procedure described for other compounds [33]. After the completion of the reaction (TLC monitoring using dichloromethane-methanol, 9.5:0.5, v/v, UV light at 254 nm) the mixture was neutralized with saturated solution of sodium carbonate, diluted with dichloromethane and then filtered. The organic layer was dried with MgSO_4_, filtered and concentrated at rotary evaporator. The residue was precipitated with ethyl ether. 50/50 racemic mixture. Yield: 90%, m.p. 165–167 °C; IR (ZnSe crystal, cm^−1^): 3362 (NH_2_), 3224 (-NH-), 2953 (CH_Ar_), 1710 (C=O, thiazolidine-4-one), 1672 (-CO-NH-), 1214 (C-N), 658 (C-S); ^1^H-NMR (δ ppm): 10.13 (brs, 1H, -NH-), 7.12 (d, *J* =7.8 Hz, 1H, Ar-H), 7.09–7.00 (m, 4H, Ar-H), 6.86 (d, *J* = 8.4 Hz, 1H, Ar-H), 6.55 (d, *J* = 7.8 Hz, 1H, Ar-H), 6.44 (d, *J* = 8.4 Hz, 1H, Ar-H), 5.66/5.46 (s, 1H, CH thiazolidine-4-one), 5.26 (d, *J* = 22.10 Hz, 2H, -NH_2_), 3.86–3.61 (m, 2H, CH_2_ thiazolidine-4-one), 3.60–3.48 (m, 1H, -CH-CH_3_), 2.41 (t, *J* = 6.8 Hz, 2H, ‑CH_2_-CH(CH_3_)_2_), 1.90–1.72 (m, 1H, -CH_2_-CH(CH_3_)_2_), 1.26 (dd, *J* = 22.4, 7.0 Hz, 3H, -CH-CH_3_), 0.86 (t, *J* = 6.0 Hz, 6H, -CH_2_-CH(CH_3_)_2_); ^13^C-NMR (δ ppm): 172.12 (C_q_), 168.69/168.47 (C_q_), 149.68/149.56 (C_q_), 138.10/137.97 (C_q_), 129.03 (CH_Ar_), 128.69 (CH_Ar_), 127.07/126.81 (CH_Ar_), 123.58 (C_q_), 113.49/113.36 (C_q_), 61.80 (CH thiazolidine-4-one), 44.25 (-CH_2_-CH(CH_3_)_2_), 42.09/41.97 (-CH-CH_3_), 29.62 (-CH(CH_3_)_2_), 29.39 (CH_2_ thiazolidine-4-one), 22.18 (-CH(CH_3_)_2_), 18.89/17.80 (-CH-CH_3_); HRMS (EI-MS): *m/z* calculated 398.189675; [M+H]^+^ found 398.189802; Green chemistry metrics: E-factor 38.478 ME 0.025.

#### 3.2.4. Preparation of 2-(4-Acetamidophenyl)-3-[2-(4-(isobutyl)phenyl)-2-methyl]acetamidothiazol-idine-4-one (**4n**)

To a solution of 2-(4-aminophenyl)-3-[2-(4-(isobutyl)phenyl)-2-methyl]acetamidothiazolidine-4-one (**4m**, 0.8 mmol) in DMF (5 mL) which was cooled at 0 °C there was added sodium hydride (0.96 mmol, 60% dispersion in mineral oil), according to the procedure described for other compounds [34]. After the mixture of reaction was stirred 1 h at room temperature under argon atmosphere, acetyl chloride (1.6 mmol) was added under low temperature (0 °C), and then the mixture was heated under reflux for 14 h. After the completion of reaction (TLC monitoring using dichloromethane-methanol, 9.5:0.5, v/v, UV light at 254 nm) the solvent was removed under reduced pressure and the residue was treated with dichloromethane and washed three times with distilled water. The organic layer was dried with MgSO_4_, filtered and concentrated at rotary evaporator. The residue was precipitated with ethyl ether. 50/50 racemic mixture. Yield: 47%, m.p. 230 °C; IR (ZnSe crystal, cm^−1^): 3340, 3206 (-NH-), 2955 (CH_Ar_), 1715 (C=O, thiazolidine-4-one), 1672 (-CO-NH-), 1212 (C-N), 659 (C-S); ^1^H-NMR (δ ppm): 10.19 (brs, 1H, -NH-), 10.01 (d, *J* = 23.2 Hz, 1H, -NH*-*CO-CH3), 7.59 (d, *J* = 8.5 Hz, 1H, Ar-H), 7.49 (d, *J* = 8.4 Hz, 1H, Ar-H), 7.34 (d, *J* = 8.5 Hz, 1H, Ar-H), 7.13 (d, *J* = 8.4 Hz, 1H, Ar-H), 7.07 (d, *J* = 7.9 Hz, 1H, Ar-H), 7.00 (dd, *J* = 11.4, 7.9 Hz, 3H, Ar-H), 5.75/5.60 (s, 1H, CH thiazolidine-4-one), 3.89–3.76 (m, 1H, CH_2_ thiazolidine-4-one), 3.70 (dd, *J* = 15.9, 7.0 Hz, 1H, CH_2_ thiazolidine-4-one), 3.59–3.45 (m, 1H, -CH-CH_3_), 2.39 (dd, *J* = 7.0, 3.7 Hz, 2H, -CH_2_-CH(CH_3_)_2_), 2.05 (d, *J* = 7.1 Hz, 3H, -NH-CO-CH3), 1.85–1.73 (m, 1H, -CH_2_-CH(CH_3_)_2_), 1.24 (dd, *J* = 22.3, 7.0 Hz, 3H, -CH-CH_3_), 0.84 (d, *J* = 6.6 Hz, 6H, -CH_2_-CH(CH_3_)_2_); ^13^C-NMR (δ ppm): 172.16 (C_q_), 168.75/168.52 (C_q_), 168.33 (C_q_), 140.01/139.92 (C_q_), 139.32 (C_q_), 138.09/137.93 (C_q_), 132.08/131.91 (C_q_), 128.70 (CH_Ar_), 128.43 (CH_Ar_), 126.98/126.74 (CH_Ar_), 118.79 (CH_Ar_), 61.30/61.12 (CH thiazolidine-4-one), 44.24 (-CH_2_-CH(CH_3_)_2_), 42.12 (-CH-CH_3_), 29.59 (-CH(CH_3_)_2_), 29.31 (CH_2_ thiazolidine-4-one), 24.01 (-NH-CO-CH_3_) 22.17 (-CH(CH_3_)_2_), 18.80/17.79 (-CH-CH_3_); HRMS (EI-MS): *m/z* calculated 440.200239; [M+H]^+^ found 440.200178; Green chemistry metrics: E-factor 33.52, ME 0.029.

### 3.3. Biological Evaluation

The antioxidant activity was estimated using the following *in vitro* tests: total antioxidant activity, DPPH and ABTS radical scavenging assays.

#### 3.3.1. Total Antioxidant Activity

The total antioxidant activity of the tested compounds was evaluated using the phosphomolybdenum method according to the procedure in [26] with minor modifications. The method is based on the reduction of Mo(VI) to Mo(V) by the tested compounds followed by the formation of a green phosphate/Mo(V) complex at acid pH. Samples of different concentrations in DMSO (2.5, 1.25, 0.625, 0.3125 mg/mL) have been used. The samples (200 µL) were mixed with the reagent solution (2 mL, 28 mM sodium phosphate; 4 mM ammonium molybdate; 0.6 M sulphuric acid), incubated at 95 °C for 90 min, cooled at room temperature. The absorbance of the samples was measured at 695 nm against a blank solution (DMSO mixed with the reagents). For each sample the effective concentration (EC_50_) was calculated. Ibuprofen and vitamin E (α-tocopherol) were used as reference and positive control, respectively. All tests were performed in triplicate.

#### 3.3.2. DPPH Radical Scavenging Assay

The radical scavenging activity of the tested compounds towards the radical 1,1-diphenyl-2-picrylhydrazyl (DPPH) was measured as described in [27] with slight modifications. DPPH in methanol (4 mL, 15 µM) was added to the test compounds (100 µL) prepared from stock solution (20 mg/mL). The concentration of the tested compounds in the sample was 487.8 µg/mL. The mixture was left for 1 h at room temperature in the dark and after that, the absorbance was measured at 517 nm against a blank solution (methanol). The radical scavenging capacity was calculated according to the following equation: Scavenging activity % = (A_control_ − A_sample_/A_control_) × 100. A_sample_ is the absorbance of the sample after 1 h. A_control_ is the absorbance of mixture of 100 µL DMSO and 4 mL DPPH). The ibuprofen and vitamin E (α-tocopherol) were used as reference and positive control, respectively. All tests were performed in triplicate.

#### 3.3.3. ABTS Radical Scavenging Assay

The ABTS**^+^** radicals were activated by reacting of ABTS (2,2'-azino-bis(3-ethylbenzthiazoline-6-sulphonic acid) (7 mM) with ammonium persulphate (2.45 mM). The mixture was left at room temperature for 16 h in the dark as described in [28]. The intensely colored ABTS^+^ radical cation solution was diluted with ethanol to obtain an absorbance value of 0.7 ± 0.02 at 734 nm. To test compound solutions (20 µL) prepared from stock solution (20 mg/mL) ABTS solution (1980 µL) was added. The concentration of the tested compounds in the sample was 200 µg/mL. After 6 min the absorbance was measured and the radical scavenging capacity was calculated according to the following equation: Scavenging activity % = (A_t__ =__ 0_ − A_t__ =__ 6min_/A_t__ =__ 0_) × 100. A_t__ =__ 0_ is the absorbance before adding the sample. A_t=6 min_ is the absorbance after 6 min of reaction. The ibuprofen and vitamin E (α-tocopherol) were used as reference and positive control respectively. All tests were performed in triplicate.

#### 3.3.4. Statistical Analysis

All antioxidant assays were carried out in triplicate. Data were analyzed by an analysis of variance (ANOVA) (*p* < 0.05) and were expressed as means ± SD. The EC50 values were calculated by linear interpolation between the values registered above and below 50% activity.

## 4. Conclusions

In this study new heterocyclic compounds that combine the thiazolidine-4-one structure with the arylpropionic acid one have been synthesized. The structure of the new compounds was proved using spectral methods (IR, ^1^H-NMR, ^13^C-NMR, MS). The compounds were evaluated for their antioxidant effects using *in vitro* assays: total antioxidant activity, DPPH and ABTS radical scavenging ability. All thiazolidin-4-one derivatives **4a**–**n** showed improved antioxidant effects in comparison with the corresponding acyl hydrazones **3a**–**l** and ibuprofen, the parent compound. The encouraging preliminary results illustrate the antioxidant potential of the synthesized compounds and motivate our next research focused on their anti-inflammatory effects in chronic and acute inflammation models.
